# Who Are the Philanthropists?

**Published:** 1891-01-03

**Authors:** 


### The Hospital

A Weekly Journal of

**Science, Medicine, Nursing, and Philanthropy.**

*For Hospitals, Asylums, Infirmaries, Dispensaries, Medical Practitioners, Students, Nurses, and the Charitable Public.*

Vol. IX.—No. 223.] [January 3, 1891.

### Who are the Philanthropists?

Every man is either a philanthropist, a misanthropist, or an indifferentist: but no man with a normal cerebrum is an indifferentist; therefore, every sane man is either a philanthropist, or a misanthropist. But is a really sane, wholesome, and hearty man ever a misanthropist? Certainly not! So we are shut up to the conclusion that every normal man is a philanthropist. This conclusion will bear thinking about. Whoever is surprised at it, or is inclined to thrust it away with contemptuous incredulity, will do well to first put it into the scales of experience and careful observation, and see whether it is not much more solid than he is inclined to think it.

There are degrees of precedence among the sciences, exactly as there are ranks and dignities among men. The science—or is it partly an art?—of forming sound judgments about the world, and men and affairs, is one of the greatest princes, if it be not the very king, of all the sciences. This also will bear thinking about. We men and women are not brute beasts, are we? We have a brain which separates us by a long interval from them. What is the particular function of our particular brain? Our business is to see, to know, to understand! Not to see what is on the surface only: a sparrow or a goose can do that: but to see down into, and through a thing; to weigh it, to measure it, to compare it with other things; to give it an exact definition, and a proper label; and to place it with due accuracy on its own convenient shelf in the warehouse of our mental merchandise.

Looking at, and judging among human affairs like men of original mind, and carefully avoiding anything like the conduct of the parrot, whose business it is to repeat with nice exactitude the very words and tones of his tutor, what is the place we assign to philanthropy among the effective faculties of civilised man? We say "civilised man," for philanthropy is one of the distinguishing marks of civilisation; and that, on a Darwinian hypothesis, is a fact of much significance. Perhaps before assigning to philanthropy its place among the effective faculties of civilised man, we had better define what it is. Because if the writer be attaching one meaning to the word, and his readers half-a-dozen other meanings, all different, there will not be much chance of agreement at the end of the article. What is philanthropy? To the reader who has as much Greek as will enable him to recall that "philein" is the verb to love, and that "anthropos" is a man, a fairly just definition of philanthropy lies ready to hand. But our definition is even simpler than that; it is, "Do as you would be done by." Here we speak of a person who is a "doer," and of another who is a "done by." The "done by" man universally likes to be treated, not only with justice, but with kindness and consideration; and as every man and woman, even the Queen and the Prime Minister, is sometimes in the position of the "done by" person, we cannot avoid the conclusion that every man, in his heart of hearts, and at the psychological moment, is emphatically a philanthropist.

If we be all philanthropists—and no man of culture, much less a man of science, will despise severe logic—it is as well that we should acknowledge the fact, both to ourselves and to the world. There is no use in fighting under false colours, or in being stored away in the human warehouse under a lying label. We have all discovered ourselves to be honourable men and philanthropists; we will stand forth and look one another in the face, and ask—"What next?" What says the poor man, who has found out his philanthropy? He says he thinks he has done very well by his neighbours and the world in being content with such an exceedingly small share of the good things that are going. His clothes are not only somewhat out of date as regards the fashion of them, but in these unusual seasons he finds that they make a poor fight against the cold. His house is not large, nor very clean, nor is the pantry at all unduly filled. The fire in the grate is of the smallest, and the blankets at night are decidedly thinner than is at all comfortable. Besides, he has not claimed much mental furniture for himself or his children. Like his house and his clothes, his mind is a little out of date and out at elbows, and somewhat sparsely furnished. Of honour—which so many of us greedily hunger after—he he does not know even the taste. As Carlyle says, no man does him reverence. When the kicks and the halfpennies are flying about, he gets as full a share of the former as of the latter; sometimes a larger share. On the whole, the poor man considers he has not done badly in the philanthropic way; in that he has not only tried to be content with a minimum of good things for himself, but has done a good deal towards helping the rich to obtain, to preserve, and to enjoy their maximum.

There is much to be said for such a line of reasoning. We will undertake to convert any Crœsus in Europe to this particular view of things in six months. All that we have to do is to "Suppose a change of cases," as Burns has it. Is any rich simpleton going to hate or to despise his poor neighbour for taking his small share so quietly? The rich man is not a philosopher if he does! If every poor toiler were as discontented, as greedy, as reckless, as insatiable in insisting upon doing and getting everything that pleases himself, as some of the rich, we might all commit suicide at once, for not only would life in this world be hateful, but it would be impossible. The poor man's philanthropy, in taking his small share of the world's prosperity so patiently, is a thing not to be despised, but rather to be acknowledged, and to be grateful for. Any rich man, indeed, who has a reasonable regard for fairness, will look upon it as an obligation, as a debt incurred by himself, which he will gratefully and honourably discharge.

It has been more than once hinted to the writer that there are here and there to be found medical men who "do not believe in philanthropy." Every medical man is a philanthropist: he cannot help it. He may be a bear, but he is a philanthropist; he may be a hungry shark, but he is a philanthropist. He cannot avoid sometimes seeing a patient for one guinea instead of two, or, if he be in a poor district, for eighteen-pence instead of half-a-crown. He is then a philanthropist to the tune of a guinea, or at the very least of a shilling. We would suggest to him that it would be better to sacrifice the guinea or the shilling sweetly, like a good-natured fellow, than to snap and snarl out his hatred of philanthropy. When he sees in-patients, or even out-patients, at the hospital, he does them good, and enjoys it. Ah, but he does not do it for their sakes but for his own, he will tell us. He hates philanthropy and believes only in self-interest. Poor man, we do not believe a single word he says. We know him to be a philanthropist; and are only sorry that he will not warm his cold heart at the fire of his own good deeds. The doctor who says he is not a philanthropist and does not believe in philanthropy, is merely a big St. Bernard, with a bear's skin on. He growls as if he would eat up the whole hospital, or parish; but the truth is that he would not hurt a hair of the smallest patient's head. Put off your bear's skin, faithful dog, and cease your growling! Nature which was too strong for Melancthon in one particular, is too strong for you in another. She made you a philanthropist; be thankful to her and have done with your half-hearted and wholly futile protests. You cannot help your destiny. Much as nature lets you see of her inner mysteries, she permits no interference at all with her fixed and changeless purposes.

Philanthropy is named as one of the marks of high intellectual development by Darwin. The least intellectual nations have the strongest regard for *meum*. As intellect advances *tuum* begins to emerge, and to be recognised. Darwin is one of your patron saints, is he not, man of science and professor of the gospel of self interest? Why, then, do you not take the trouble to understand the rudiments af your own faith? The truth is that we all have the will to do a good turn to our fellow-men when opportunity occurs. Men sometimes get a little soured by the weakness and wickedness of some of the poorest classes. But the wholesome wine of human kindness never remains permanently sour. In spite of ourselves and our head theories, our hearts are still warm human organs that must sometimes act on the impulsion of kindness and generosity alone. We take our stand on this—on Nature. Whatever is universally natural must, when rightly understood, be universally right. It is right to help the needy; it is right to soothe the sorrowing; it is right to heal the sick; and most right of all is it to succour, without a moment's delay, those who are, at one and the same time, needy and sorrowful and sick. Whosoever is convinced by our reasoning, let him give effect to his convictions in prompt and open-handed practice.

